# Changes in pre- and postoperative serum leptin concentrations in dogs with gallbladder mucocele and cholelithiasis

**DOI:** 10.1186/s12917-019-1964-z

**Published:** 2019-06-25

**Authors:** Sungin Lee, Aeri Lee, Oh-kyeong Kweon, Wan Hee Kim

**Affiliations:** Department of Veterinary Clinical Sciences College of Veterinary Medicine and Research Institute for Veterinary Science, Seoul National University, 1 Gwanak-ro, Gwanak-gu, Seoul, 151-742 Republic of Korea

**Keywords:** Canine, Cholecystectomy, Cholelithiasis, Gallbladder mucocele, Leptin

## Abstract

**Background:**

Leptin has been shown to have various physiological and pathological roles in the canine gallbladder. In this study, we performed pre- and postoperative short-term follow-up analyses to confirm changes in serum leptin levels before and after cholecystectomy due to gallbladder mucocele (GBM) or cholelithiasis in dogs.

**Results:**

Twenty-six cholecystectomized dogs (GBM: *n* = 14; cholelithiasis: *n* = 12) for prophylactic or clinical symptom relief were enrolled in the present study. Dogs were subgrouped according to clinical symptoms and prognosis after surgery as follows: 1) asymptomatic group (*n* = 13), 2) recovery group (*n* = 8), and 3) death group (*n* = 5). Liver enzymes, total bilirubin, lipid profiles, and leptin concentrations were determined from sera on the pre-operative day and at 1, 3, and 7 days postoperation. Serum leptin concentrations were gradually but significantly decreased in the asymptomatic group (*p* = 0.008, 0.004, and 0.004 on days 1, 3, and 7, respectively, compared with that before surgery) and the recovery group (*p* = 0.048 and 0.048 on days 3 and 7, respectively, compared with that before surgery). However, in the death group, leptin concentrations did not differ significantly over time (*p* = 0.564). Additionally, serum leptin levels in the recovery group (*p* = 0.006) and death group (*p* = 0.021) were significantly higher than those in the asymptomatic group. Liver enzymes and total bilirubin (T-Bil) were significantly decreased only in the recovery group, particularly on day 7. In the asymptomatic group, liver enzymes and T-Bil were not changed significantly over time, and in the death group, only T-Bil was significantly decreased on day 7. Total cholesterol and triglyceride levels were not significantly decreased over time in all groups.

**Conclusions:**

These results indicate that leptin is a potential biomarker reflecting the severity and prognosis of GBM and cholelithiasis both before and after cholecystectomy in dogs.

## Background

Extrahepatic biliary tract obstruction (EHBO) is a life-threatening condition owing to the interruption of bile flow, which causes severe physiological problems and can lead to death. Among various pathological conditions that obstruct bile flow, gallbladder mucocele (GBM) and cholelithiasis are important causes of EHBO in dogs, necessitating surgical intervention [1]. The incidence and diagnostic rate of GBM and cholelithiasis have recently increased, and the mortality rate associated with EHBO surgery is still high [2]. Although the cause and etiopathogenesis of GBM and cholelithiasis in dogs have not been fully elucidated, previous studies have suggested that GBM formation is related to gene mutations [3], endocrinopathies [4], and hyperlipidemia [5], and pathogenesis of cholelithiasis is associated with gallbladder motility [6] and alterations of absorption and secretion functions in the gallbladder [7].

Leptin is a peptide hormone predominantly synthesized in adipose cells that functions to regulate the energy balance by inhibiting hunger signals from the hypothalamus [8]. After the discovery of leptin, many studies evaluated the relationships between leptin and biological mechanisms related to obesity [9–11]. In addition, leptin has been shown to have multifunctional physiological roles in various organs, including the brain [12], skeletal muscle [13], vasculature [14], heart [15], gastrointestinal tract [16], and liver [17]. Furthermore, alterations in the levels of adipokines, including leptin, have highlighted the potential applications of these molecules as biomarkers and therapeutic targets in various obesity-related diseases and neoplasias in human medicine [18].

Many previous studies have suggested that there may be a relationship between leptin and the gallbladder. Indeed, leptin has been shown to regulate gallbladder gene expression and motility [7, 19]. Moreover, we previously evaluated the relationship between leptin and gallbladder disease in dogs. We demonstrated that leptin and leptin receptor are expressed in the canine gallbladder, indicating that the gallbladder is a source of leptin and can be affected by leptin [20]. Furthermore, the expression levels of leptin and its receptor are increased in the gallbladder in canine patients with GBM and cholelithiasis, and serum leptin concentrations are also significantly increased compared with those in normal healthy dogs. In particular, the severity of clinical signs and the systemic state of patients is associated with serum leptin concentrations [21, 22].

In this study, we aimed to examine serum leptin concentrations and serum biochemical parameters in patients with GBM or cholelithiasis before and after surgery and investigated changes in leptin concentrations over time.

## Results

### Cases

In total, 26 dogs that underwent cholecystectomy for GBM (*n* = 14) and cholelithiasis (*n* = 12) were studied. The enrolled dogs were categorized into the asymptomatic, recovery, and death groups. Detailed demographic characteristics are summarized in Table 1. The mean body weights of the dogs in three groups were 4.68, 4.43, and 5.1 kg, respectively, and there were no significant differences among groups (*p* = 0.783). BCS and age did not significantly differ between the three groups (*p* = 0.0.441 and *p* = 0.666, respectively). Eight of the 14 dogs with GBM (57.14%) and five of the 12 dogs with cholelithiasis (41.67%) were confirmed as having EHBO on imaging tests and exhibited related clinical signs, including anorexia, vomiting, icterus, and abdominal pain. When operation was performed due to clinical symptoms caused by EHBO, three of the eight dogs with GBM (37.5%) and two of the five dogs with cholelithiasis (40%) died without recovering. Of 14 dogs with GBM and 12 dogs with cholelithiasis, eight (57.14%) and seven (58.33%) exhibited hyperlipidemia.

**Table 1:** Demographic characteristics of the study population

	Asymptomatic group	Recovery group	Death group
Age (years)	13 (5)	12 (2)	11 (3)
Sex (n)	Female (2)	Female (1)	Female (2)
	Male (1)	Spayed female (3)	Spayed female (2)
	Spayed female (3)	Castrated male (4)	Castrated male (1)
	Castrated male (7)		
Breed (n)	Cocker Spaniel (1)	Dachshund (1)	Cocker Spaniel (1)
	Maltese (2)	Maltese (1)	Dachshund (1)
	Mixed breed (1)	Mixed breed (2)	Maltese (1)
	Pomeranian (1)	Pomeranian (1)	Pomeranian (1)
	Poodle (1)	Poodle (1)	Yorkshire Terrier (1)
	Schnauzer (1)	Shih Tzu (2)	
	Shih Tzu (4)		
	Yorkshire Terrier(2)		
BW (kg)	4.68 ± 1.44	4.43 ± 1.32	5.1 ± 2.13
BCS (/9)	5 (5–6)	5 (5–6)	6 (5–6)

Continuous variables are presented as means and standard deviations, except for age and sex (which are presented as the median followed by the interquartile range). BCS, which is a discontinuous value, is reported as the median and range. ^*^Significant difference (*p* < 0.05) between the three groups. *BCS*, body condition score; *BW*, body weight; *GBM*, gallbladder mucocele; *n*, number of patients

### Analysis of serum leptin, TG, T-Chol, T-Bil, and liver enzyme profiles

**Fig. 1:**
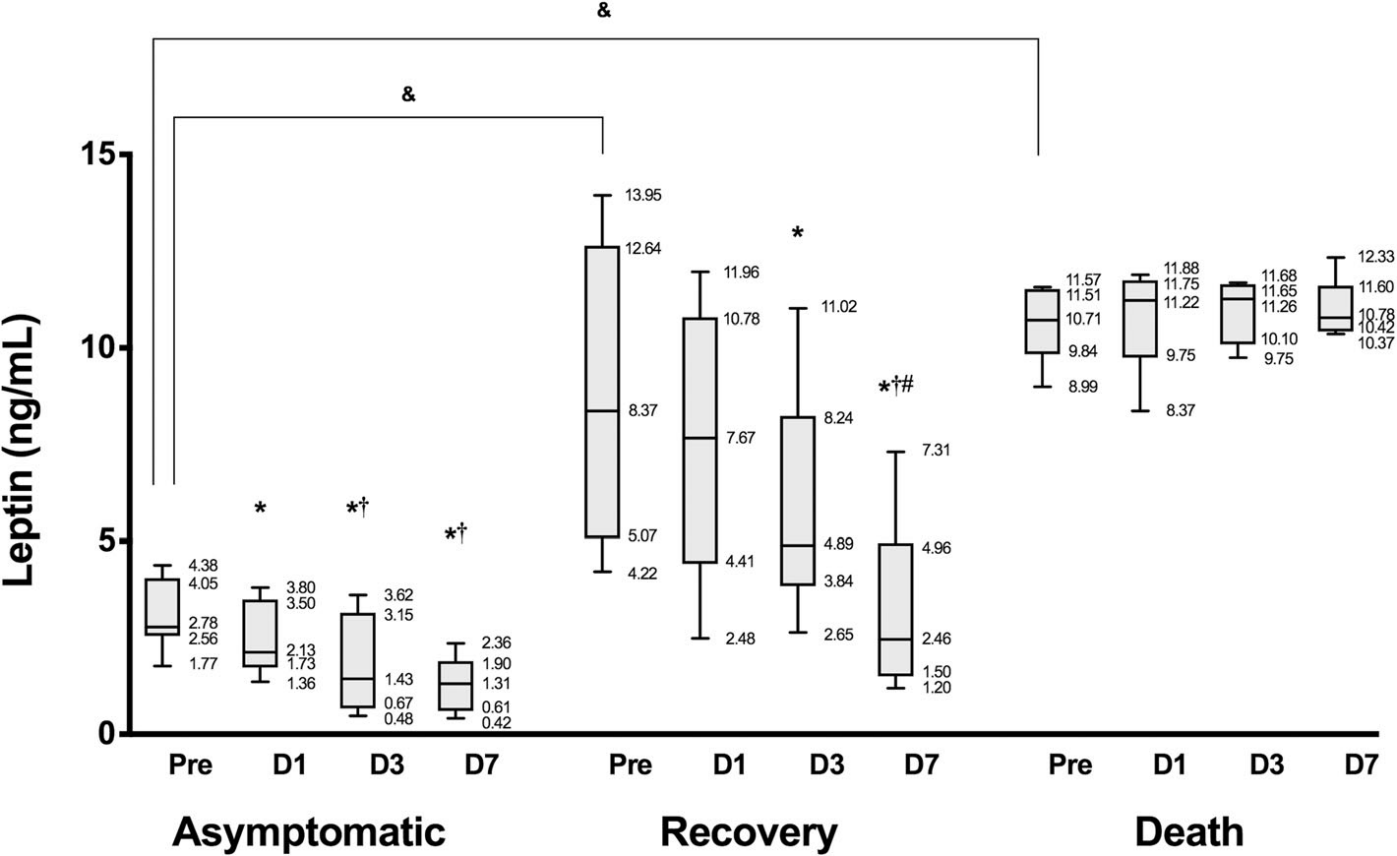
Changes in serum leptin concentrations over time. Horizontal bars in each box plot indicate median values. ^*^Significant difference (*p* < 0.05) compared with pre in each group. ^†^Significant difference (*p* < 0.05) compared with D1 in each group. ^#^Significant difference (*p* < 0.05) compared with D3 in each group. ^&^Significant difference (*p* < 0.05) between three groups at pre. *Pre*, pre-operative day 1; *D1*, postoperative day 1; *D3*, postoperative day 3; *D7*, postoperative day 7

Serum leptin concentrations are shown in Fig. 1. Most dogs that underwent cholecystectomy, except those in the death group, showed decreased serum leptin concentrations over time. In the asymptomatic group, relative to that on pre-operative day 1, leptin concentrations were decreased significantly on postoperative days 1–7 (*p* = 0.008, 0.004, and 0.004, respectively). Additionally, relative to that on postoperative day 1, leptin concentrations were decreased significantly on postoperative days 3 and 7 (*p* = 0.008, 0.004, respectively). In the recovery group, serum leptin levels were significantly decreased on postoperative days 3 and 7 compared with that before surgery (*p* = 0.048, 0.048, respectively) and were significantly decreased on postoperative day 7 compared with those on postoperative days 1 (*p* = 0.048) and 3 (*p* = 0.048). In the death group, leptin levels did not differ significantly over time (*p* = 0.564). Moreover, pre-operative leptin levels were significantly higher in the recovery group (*p* = 0.006) and death group (*p* = 0.021) than in the asymptomatic group, and the concentration difference was similar between the recovery and death groups (*p* = 0.564). Similarly, leptin levels were significantly higher in the recovery group (*p* = 0.006, 0.006, respectively) and death group (*p* = 0.003, 0.003, respectively) than in the asymptomatic group on postoperative days 1 and 3, and there were no significant differences between the recovery and death groups on these days (*p* = 0.321, 0.213, respectively). On day 7 postoperation, there were similar differences in concentrations between the asymptomatic and recovery groups (*p* = 0.09), whereas that in the death group was significantly higher than those in the asymptomatic group (*p* = 0.003) and recovery group (*p* = 0.009).

Detailed results of blood analyses are shown in Tables 2, 3, 4. In the asymptomatic group (Table 2), serum T-Bil (*p* = 0.141) and liver enzyme profiles (such as ALT, AST, ALP, and GGT; *p* = 0.189, 0.117, 0.557, and 0.641, respectively) were not significantly different over time. In the recovery group (Table 3), serum ALT, AST, ALP, GGT, and T-Bil levels were gradually decreased over time after surgery; in particular, all values were significantly decreased at postoperative day 7 compared with those on pre-operative day 1 and postoperative day 1 (*p* < 0.05; Table 3). In the death group (Table 4), the ALT (*p* = 0.178), AST (*p* = 0.086), ALP (*p* = 0.072), and GGT (*p* = 0.098) levels did not differ significantly between time points. However, serum T-Bil was significantly decreased on postoperative day 7 compared with that on pre-operative day 1 (*p* = 0.048), postoperative day 1 (*p* = 0.048), and postoperative day 3 (*p* = 0.048). There were no significant differences in T-Chol and TG levels in all groups (asymptomatic group, *p* = 0.059 and 0.485; recovery group, *p* = 0.93 and 0.583; death group, *p* = 0.782 and 0.186, respectively).

**Table 2:** Changes in serum TG, T-Chol, and T-Bil concentrations and liver enzyme profiles over time in dogs in the asymptomatic group

	Pre-OP	Post-OP 1	Post-OP 3	Post-OP 7
ALT (6–90 U/L)	48 (34.5)	90 (84)	87 (99)	90 (103.5)
AST (10–43 U/L)	30 (9)	37 (53)	31 (43.5)	33 (35.58)
ALP (8–100 U/L)	135 (370)	222 (460)	203 (265)	198 (236.5)
GGT (0–14 U/L)	8 (3.5)	9 (7)	12 (16)	11 (24.5)
T-Bil (0–0.6 mg/dL)	0.02 (0.05)	0.05 (0.11)	0.03 (0.07)	0.05 (0.04)
TG (21–133 mg/dL)	127 (129.5)	105 (115.5)	113 (44)	88 (43)
T-Chol (112–312 mg/dL)	290 (128)	267 (95.5)	273 (74.5)	272 (80)

Data are presented as the median and interquartile range. ^*^Significant difference (*p* < 0.05) in change over time. *ALT*, alanine aminotransferase; *AST*, aspartate aminotransferase; *ALP*, alkaline phosphatase; *GGT*, glutamyl transpeptidase; *T-Bil*, total bilirubin; *TG*, triglyceride; *T-Chol*, total cholesterol; *Pre-OP*, pre-operative day; *Post-OP*, postoperative day

**Table 3:** Changes in serum TG, T-Chol, and T-Bil concentrations and liver enzyme profiles over time in dogs in the recovery group

	Pre-OP	Post-OP 1	Post-OP 3	Post-OP 7
ALT (6–90 U/L)	1280.5 (1877.25)	853 (1023)	330 (598) *^†^	213 (259.75) *^†#^
AST (10–43 U/L)	234 (230.75)	191.5 (272.3) *	115.5 (88) *^†^	99 (160.75) *^†^
ALP (8–100 U/L)	1411.5 (2422.25)	1154 (2237.8) *	666 (1795) *^†^	477 (1536) *^†#^
GGT (0–14 U/L)	90.5 (138.25)	82.5 (176.5) *	65 (66.5) ^†^	49 (53.25) ^†#^
T-Bil (0–0.6 mg/dL)	8.74 (38.27)	3.86 (32.23)	1.58 (23.98) ^†^	0.77 (9.63) *^†#^
TG (21–133 mg/dL)	140.5 (155.5)	129.5 (64.5)	140 (25.25)	125 (15.75)
T-Chol (112–312 mg/dL)	384.5 (154.4)	366.38 (190.7)	353.4 (168.8)	359 (180.26)

Data are presented as the median and interquartile range, except for T-Chol, which is presented as the mean and standard deviation. ^*^Significant difference (*p* < 0.05) compared with Pre-OP. ^†^Significant difference (*p* < 0.05) compared with Post-OP 1. ^#^Significant difference (*p* < 0.05) compared with Post-OP 3. *ALT*, alanine aminotransferase; *AST*, aspartate aminotransferase; *ALP*, alkaline phosphatase; *GGT*, glutamyl transpeptidase; *T-Bil*, total bilirubin; *TG*, triglyceride; *T-Chol*, total cholesterol; *Pre-OP*, pre-operative day; *Post-OP*, postoperative day

**Table 4:** Changes in serum TG, T-Chol, and T-Bil concentrations and liver enzyme profiles over time in dogs in the death group

	Pre-OP	Post-OP 1	Post-OP 3	Post-OP 7
ALT (6–90 U/L)	253 (994)	312 (1030.5)	397 (824.5)	286 (517)
AST (10–43 U/L)	149 (1106.5)	134 (559.5)	67 (51.5)	71 (57.5)
ALP (8–100 U/L)	2518 (1383.24)	2243.4	1690 (888.8)	1143.8 (652.5) ^†^
GGT (0–14 U/L)	130 (131.5)	121 (101)	52 (55)	36 (89)
T-Bil (0–0.6 mg/dL)	8.52 (1.34)	4.07 (1.82) *	1.26 (1.16) *^†^	0.57 (0.38) *^†^
TG (21–133 mg/dL)	156 (147.5)	134 (166)	131 (165)	113 (191.5)
T-Chol (112–312 mg/dL)	468 (249.91)	432.2 (268.8)	439.4 (260.4)	415.8 (252.51)

Data are presented as the median and interquartile range, except for ALP, T-Bil, and T-Chol, which are presented as the mean and standard deviation. ^*^Significant difference (*p* < 0.05) compared with Pre-OP. ^†^Significant difference (*p* < 0.05) compared with Post-OP 1. *ALT*, alanine aminotransferase; *AST*, aspartate aminotransferase; *ALP*, alkaline phosphatase; *GGT*, glutamyl transpeptidase; *T-Bil*, total bilirubin; *TG*, triglyceride; *T-Chol*, total cholesterol; *Pre-OP*, pre-operative day; *Post-OP*, postoperative day

## Discussion

Previous studies have verified that serum leptin concentrations are associated with various diseases in dogs, including cardiovascular disease [15], pancreatitis [23], and hormone imbalances, such as hyperadrenocorticism [24], hypothyroidism [25], and diabetes mellitus [26]. Moreover, plasma leptin concentrations vary according to BCS, regardless of age, sex, and breed [27]. However, in this study, there were no significant differences in serum leptin concentrations with age, sex, breed, body weight, and BCS between groups. Furthermore, age, sex, breed, body weight, and BCS were not related to serum leptin levels. In order to rule out the influence of other diseases and BCS, only dogs with similar BCS (5–6/9) and no concurrent diseases except GBM and cholelithiasis were selected.

Diagnoses of GBM and cholelithiasis are made based on the comprehensive judgment of laboratory findings, abdominal radiographs, ultrasonography, and clinical symptoms, such as vomiting, anorexia, lethargy, abdominal pain, and icterus. Clinical presentations and laboratory findings in patients with GBM and cholelithiasis vary widely, and most cases are asymptomatic. The manifestation of symptoms is associated with biliary tract obstruction caused by mucoid material and choleliths. Abdominal radiographs are inadequate to definitively diagnose GBM. Instead, diagnosis of GBM and cholelithiasis is most commonly performed using abdominal ultrasonography, which has become the current gold standard in dogs [1, 28, 29]. However, despite the various diagnostic tools available, imaging results, changes in laboratory findings, and clinical signs can all vary. For this reason, it is difficult to assess the progression of the disease and prognosis. Many studies have recently examined factors that can be used to evaluate the progression and prognosis of these diseases. For example, in a previous study, to evaluate the association between circulating leptin concentration and the severity of GBM and cholelithiasis, patients in the GBM and cholelithiasis groups were separated into those that underwent surgery owing to clinical manifestations and those that did not; serum leptin concentrations were found to be significantly higher in the operated group than in the nonoperated group. These findings suggested that serum leptin concentrations may have applications as a biomarker for assessing the severity of GBM and cholelithiasis in dogs [21, 22]. The results of these previous studies are consistent with those of the present study, demonstrating that serum leptin concentrations in dogs with GBM or cholelithiasis were significantly higher in patients who underwent surgery to alleviate clinical symptoms than in those who underwent prophylactic surgery.

In humans, previous studies have investigated not only the associations between leptin and diseases requiring surgical intervention but also changes in leptin concentrations following surgery. One study showed that serum leptin concentrations were gradually decreased from 16 h after operation in patients who underwent surgery due to trauma or large bowel diseases [30]. Similar results were also observed in a study in women who underwent total abdominal hysterectomy [31]. In veterinary medicine, a study reported changes in leptin concentrations before and after treatment in dogs with naturally occurring pituitary-dependent hyperadrenocorticism. The results of showed that serum leptin levels were significantly decreased after treatment [24]. Notably, in the current study, we demonstrated that circulating leptin levels were decreased over time in all groups after surgery compared with the pre-operative level; furthermore, there were no significant changes in leptin concentrations over time in dogs that died without recovery after the operation. Taken together with the results of previous studies, our findings supported the possibility that serum leptin levels may have applications in assessing the prognosis of canine patients undergoing surgery for GBM and cholelithiasis.

The occurrence of GBM and cholelithiasis is related to various pathophysiological factors, including gallbladder motility, biliary excretion, and changes in biliary properties, and several previous studies on the direct or indirect relationships between these factors and leptin have been reported. Contraction of the gallbladder smooth muscle can be induced by neurotransmitters, such as cholecystokinin (CCK), neuropeptide Y (NPY), and acetylcholine (ACh), which exert excitatory effects on the biliary tract by stimulating the sphincter of Oddi and gallbladder contractility [19, 32, 33]. Moreover, leptin upregulates gallbladder genes, such as the genes encoding CCK A receptor, ACh B2 receptor, and NPY receptor 1, associated with hormone- and neurotransmitter-mediated gallbladder motility [34]. Other studies have shown that gallbladder responses to increasing concentrations of ACh, NPY, and CCK-8 are decreased in leptin-deficient obese mice compared with that in lean mice [19]. These results suggested that leptin may promote the motility of the gallbladder and increase bile excretion. In another study, leptin was shown to be involved in changes in biliary properties by regulating water, cation, chloride, and sodium ion transport genes related to secretion/reabsorption as follows: upregulation of aquaporin 1 and sodium-potassium-chloride cotransporter; and downregulation of aquaporin 4, Na^+^-K^+^-ATPase α1 polypeptide, chloride channel 5, and epithelial sodium channel α. Therefore, these results indicated that biliary properties may be altered due to dysregulation of leptin [7].

Our present study showed that there were no significant changes in liver enzymes and bilirubin before and after surgery in the asymptomatic group. This is probably because most patients in the symptom-free group did not also exhibit increased liver enzymes and bilirubin before surgery. In patients with EHBO, both in the recovery group and the death group, significant elevations in pre-operative liver enzymes and bilirubin levels were confirmed, and decreases in these parameters were observed after surgery. However, no significant decreases in liver enzymes or bilirubin were observed in the death group, possibly because of the small number of samples. In the EHBO state, biliary stasis, hepatic necrosis, and hemolysis were suggested as possible causes of elevations in liver enzymes and bilirubin [35], and resolution of the EHBO condition results in decreased liver enzymes and bilirubin levels [35, 36]. In this study, liver enzyme values were not significantly different between dogs in the recovery group and those in the death group before and after surgery. However, in a previous study, liver enzymes were high in dogs with a poor prognosis [37]. These contradictory results regarding liver enzymes and prognosis suggest that low levels of liver enzymes and bilirubin may be used to assess the severity or prognosis of biliary disease. Therefore, further studies are needed to evaluate the clear correlations among these factors.

Hyperlipidemia refers to a state in which the degradation of lipoprotein is delayed or in which lipoprotein is rapidly produced, even after fasting for 10 h or more; under these conditions, T-Chol (hypercholesterolemia) and TG (hypertriglyceridemia) are increased in the blood [38]. One retrospective case control study showed that the odds ratios (ORs) for GBM were 2.92- and 3.55-fold higher in dogs with hypercholesterolemia (15/36 cases) or hypertriglyceridemia (13/24 cases), respectively, than in those without these conditions [5]. Another study suggested that the ORs of cholelithiasis in dogs with hypercholesterolemia (9/43 cases) or hypertriglyceridemia (11/34 cases) were 9.72- and 12.91-fold higher, respectively, than those in dogs without hyperlipidemia [21]. These results of previous studies indicated that hyperlipidemia was associated with the pathogenesis of GBM and cholelithiasis, consistent with results of present study. In additional, serum cholesterol and TG concentrations were associated with gallbladder responses to neurotransmitters, such as CCK, NPY, and ACh. Increased serum levels of cholesterol and TG were associated with reduced sensitivity to CCK, NPY, and ACh, resulting in gallbladder dysmotility [39, 40].

The present study had several limitations. First, there was a relatively small number of patients with GBM and cholelithiasis included in this study. Analyzing a large number of patients would help to reach more reasonable and rational outcomes, and multidimensional analysis could be possible. Moreover, the number of patients was too small to allow for subgrouping according to disease. Therefore, performing independent analyses in each group would provide a better understanding of the associations between leptin and these conditions. Second, this research was conducted on patients recruited from SNU Veterinary Medical Teaching Hospital rather than on experimental animals, and all dogs participating in this study underwent history review, physical examination, urinalysis, blood analysis, radiography, and ultrasonography. When other underlying diseases such as endocrinopathies were suspected through review of the abovementioned laboratory tests, additional examinations were conducted for differential diagnosis. However, when basic laboratory results showed no abnormalities, no additional tests were performed. Since hormonal assessment was not carried out in all patients, there is the possibility that patients with overt hormonal abnormalities without clinical signs were included in this study, and this could have had an effect on the observed differences in serum leptin concentrations. Third, this study was carried out using short-term follow-up results. Thus, long-term follow-up studies on the changes in leptin levels in the peri-operative phase are needed. Finally, although the present study focused on the relationship between leptin concentrations and cholecystectomy in GBM and cholelithiasis, other gallbladder diseases requiring surgical interventions, such as gallbladder tumor, cholecystitis, and EHBO caused by pancreatitis, should be included in further studies. These additional analyses may improve our understanding of the relationships between leptin and cholecystectomy.

## Conclusion

Our pre- and postoperative short-term follow-up study revealed that serum leptin concentrations were associated with the severity of disease and clinical signs both before and after surgery in dogs with GBM and cholelithiasis. These results suggest that serum leptin concentrations may be evaluated as a prognostic factor in patients with GBM and cholelithiasis after cholecystectomy. In addition, our findings confirmed the potential application of serum leptin levels as a parameter to assess the severity of GBM and cholelithiasis before surgery. However, further studies including larger cohorts are required to definitively elucidate the relationship between leptin and gallbladder diseases, such as GBM and cholelithiasis, in dogs.

## Methods

### Sample preparation

**Fig. 2:**
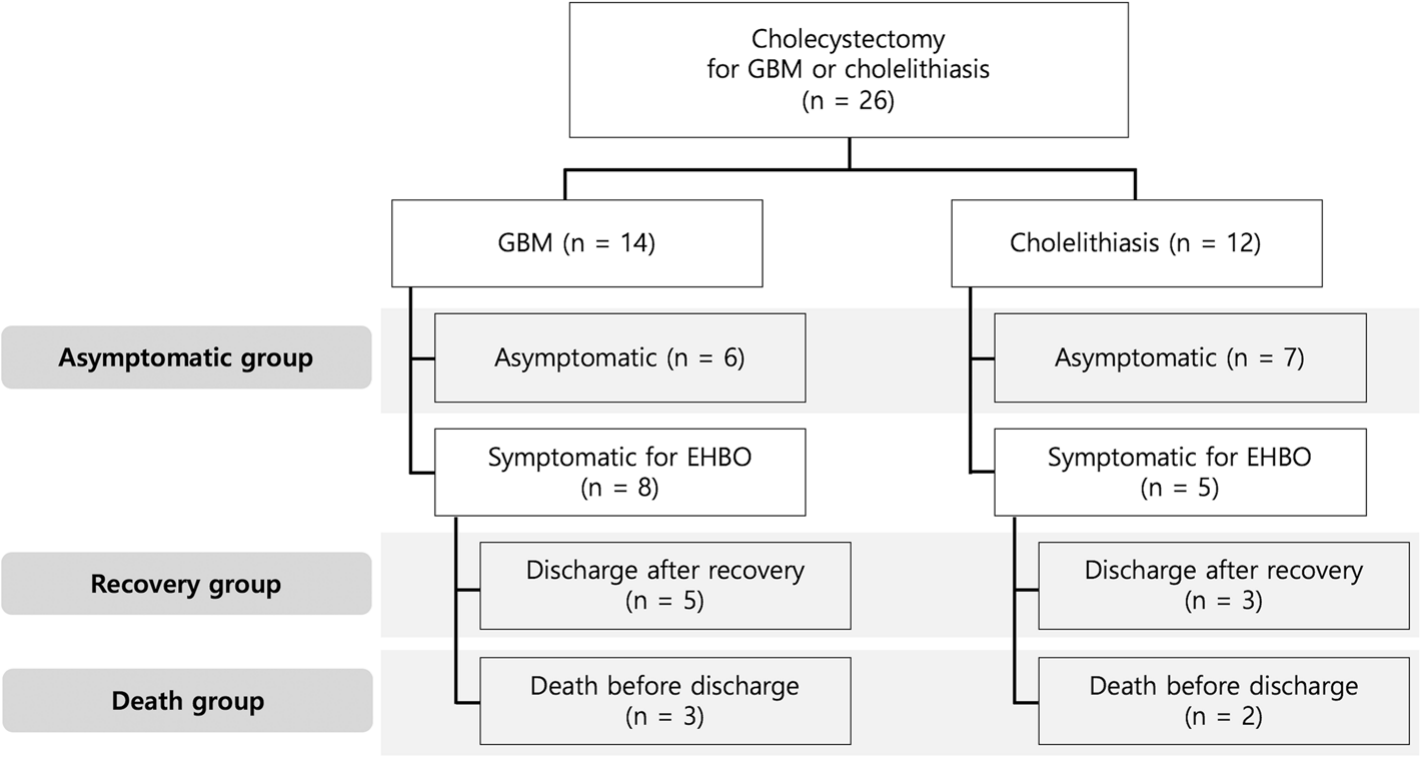
Patient classification diagram. GBM, gallbladder mucocele; EHBO, extrahepatic biliary tract obstruction; n, number of patients

Client-owned dogs with newly diagnosed GBM (*n* = 14) and cholelithiasis (*n* = 12) recruited from SNU Veterinary Medical Teaching Hospital were enrolled in this study. Only dogs with similar body condition scores (BCSs) and no concurrent diseases except GBM and cholelithiasis were selected. All dogs participating in this study underwent physical examination, including BCS, urinalysis, complete blood cell counts, serum chemistry analysis, radiography (abdominal and thoracic), and ultrasonography. All measurements of BCS were performed by the same investigator using a 9-point scale system [41]. The diagnosis of GBM and cholelithiasis were made according to criteria previously described using abdominal radiography and ultrasonography at the Department of Veterinary Radiology, College of Veterinary Medicine, SNU [28, 42, 43]. All dogs that participated in this study underwent cholecystectomy for prophylactic or clinical symptom relief and were categorized into three groups to evaluate changes before and after surgery: 1) asymptomatic group (*n* = 13), in which dogs did not have symptoms but underwent preventive surgery; 2) recovery group (*n* = 8), in which dogs recovered after cholecystectomy due to clinical symptoms caused by EHBO; and 3) death group (*n* = 5), in which dogs did not recover after cholecystectomy due to clinical signs of EHBO. Detailed patient classification information for these dogs is presented in Fig. 2.

For biochemical analysis, blood samples were collected from the jugular vein. Before performing venipuncture, the dogs were fasted for 12 h, and blood sample collection was performed on pre-operative day 1 and on postoperative days 1, 3, and 7. For separating serum, blood was collected in serum separation tubes and centrifuged at 1000×*g* for 10 min. A portion of separated serum was used for determination of total cholesterol (T-Chol), triglyceride (TG), total bilirubin (T-Bil), and liver enzyme profiles (alkaline phosphatase [ALP], alanine aminotransferase [ALT], aspartate aminotransferase [AST], and glutamyl transpeptidase [GGT]). The remaining serum was frozen at − 80 °C until enzyme-linked immunosorbent assay (ELISA) analyses for measurement of leptin concentrations. All procedures for serum chemistry analysis were performed using a Hitachi 7180 automated biochemical analyzer (Hitachi, Tokyo, Japan) with commercial kits (JW Pharmaceutical, Seoul, Republic of Korea), spectrophotometrically.

### Sandwich ELISA

Serum leptin concentrations in all samples were measured in duplicate according to the manufacturer’s protocol using a commercial canine-specific leptin sandwich ELISA kit (Canine Leptin ELISA; Millipore, Billerica, MA, USA), where the intra- and interassay coefficients of variation were 4 and 6%, respectively. The absorbances were assessed using an automated microplate spectrophotometer (Epoch, BioTek Instruments Inc., Winooski, VT, USA) at 450 nm.

### Statistical analysis

The data were analyzed using SPSS software, version 23.0 (SPSS Inc., Chicago, IL, USA). The Shapiro-Wilk test was performed to evaluate whether the data had a normal distribution. One-way analysis of variance (ANOVA) was used to analyze differences in body weights between the three groups (asymptomatic group, recovery group, and death group), and data are presented as the means and standard deviations (SDs). When age, sex, and BCS did not follow a normal distribution, the analysis was performed using Kruskal-Wallis tests. Age was presented as the median and interquartile range, and BCS, which is a discontinuous variable, was presented as the median and range. The Friedman test was performed in order to evaluate differences in serum leptin, TG, T-Chol, and T-Bil concentrations and in liver enzyme profiles over time (pre-operative day 1 and postoperative days 1, 3, and 7) in each of the three groups (asymptomatic group, recovery group, and death group), except for data with normal distributions, such as T-chol concentrations in the asymptomatic and recovery groups and ALP, T-Bil, and T-Chol concentrations in the death group. When significant differences were found, post-hoc comparisons were performed using Wilcoxon signed ranks tests with Bonferroni-Holm multiple comparison adjustments. The data are presented as median values followed by interquartile ranges. Data with normal distributions were analyzed using repeated measures ANOVA, and post-hoc tests were performed the Bonferroni-Holm method. These data were then presented as means and SDs. Differences in serum leptin concentrations between the three groups at each time point were analyzed using Kruskal-Wallis tests. When significant differences were obtained, post-hoc tests were performed using Mann-Whitney tests with Bonferroni correction. The relationships of pre-operative circulating leptin concentrations with age, sex, body weight, BCS, and breed were estimated using Spearman rank correlation tests. Results with *p* values of less than 0.05 were considered statistically significant.

## Data Availability

All relevant data are within this paper. The datasets generated and/or analyzed during the current study are available from the corresponding author on reasonable request.

## Abbreviations

ACh: Acetylcholine
ALP: Alkaline phosphatase
ALT: Alanine aminotransferase
ANOVA: one-way analysis of variance
AST: Aspartate aminotransferase
BCS: Body condition score
CCK: Cholecystokinin
EHBO: Extrahepatic biliary tract obstruction
ELISA: Enzyme-linked immunosorbent assay
GBM: Gallbladder mucocele
GGT: Glutamyl transpeptidase
NPY: Neuropeptide Y
OR: Odds ratio
SD: Standard deviation
T-Bil: Total bilirubin
T-Chol: Total cholesterol
TG: Triglyceride
